# Superior broadband antireflection from buried Mie resonator arrays for high-efficiency photovoltaics

**DOI:** 10.1038/srep08915

**Published:** 2015-03-09

**Authors:** Sihua Zhong, Yang Zeng, Zengguang Huang, Wenzhong Shen

**Affiliations:** 1 Institute of Solar Energy, and Key Laboratory of Artificial Structures and Quantum Control (Ministry of Education), Department of Physics and Astronomy, Shanghai Jiao Tong University, Shanghai 200240, People's Republic of China; 2 Collaborative Innovation Center of Advanced Microstructures, Nanjing University, Nanjing. 210093, People's Republic of China

## Abstract

Establishing reliable and efficient antireflection structures is of crucial importance for realizing high-performance optoelectronic devices such as solar cells. In this study, we provide a design guideline for buried Mie resonator arrays, which is composed of silicon nanostructures atop a silicon substrate and buried by a dielectric film, to attain a superior antireflection effect over a broadband spectral range by gaining entirely new discoveries of their antireflection behaviors. We find that the buried Mie resonator arrays mainly play a role as a transparent antireflection structure and their antireflection effect is insensitive to the nanostructure height when higher than 150 nm, which are of prominent significance for photovoltaic applications in the reduction of photoexcited carrier recombination. We further optimally combine the buried Mie resonator arrays with micron-scale textures to maximize the utilization of photons, and thus have successfully achieved an independently certified efficiency of 18.47% for the nanostructured silicon solar cells on a large-size wafer (156 mm × 156 mm).

Light reflection is undesirable for optoelectronic applications such as photodetectors, photodiodes and solar cells, since reflection leads to the loss of incident photons and thus the low generation of photocarriers. Various technologies based on different antireflection mechanisms have been developed to suppress the surface reflection. Single quarter-wavelength dielectric coating is widely used to perfectly eliminate the reflectance at a specific wavelength, the antireflection mechanism behind which is the destructive interference^1^,^2^,^3^. Micron-scale textures with morphologies including pyramid^4^,^5^ and bowl-like^6^ are also commonly utilized to suppress the surface reflectance by multiple reflections between structures. Recently, sub-wavelength structures have attracted intensive attentions due to their outstanding antireflection characteristics^7^,^8^. Excellent antireflection effects over a broad wavelength range and a wide incident angles can be achieved through appropriately designing the morphology of nanostructures to produce a gradient refractive index change from air to the substrate^9^,^10^,^11^. Meanwhile, short nanostructures^8^,^12^,^13^,^14^ as well as plasmonic nanoparticles tend to provide strong light scatterings from the excitation of resonances^15^,^16^,^17^, which also play prominent roles in light management.

However, these antireflection techniques are not ideal choices for silicon photovoltaic applications due to either the optical or electrical problems. There are still high photons loss for micron-scale textures whose surface reflectance are often higher than 10% over the whole wavelength range from 400 to 1100 nm^18^. Though the silicon nanostructures with an adiabatic refractive index change from air to substrate can effectively suppress the refection over a broad wavelength, they require high-aspect-ratio structures and thus incur extremely severe carrier recombination^19^,^20^. The antireflection mechanisms of the quarter-wavelength dielectric coatings determine that their antireflection effect depends strongly on the incident wavelength and thus the solar spectrum averaged reflectance (*R*_ave_) is still relatively high^7^. The short silicon nanostructures and plasmonic nanoparticles also only reduce the surface reflectance effectively in a narrow wavelength range^17^,^21^,^22^, indicating that they are not perfect options for the silicon solar cells that work on a broad solar spectrum either.

In this study, we provide a design guideline for an antireflection structure constituted of buried Mie resonator arrays to attain a superior broadband antireflection effect, where the role of Mie resonators is played by silicon nanostructures standing on a silicon substrate and buried under a dielectric film (see Figure 1a,b). Spinelli *et al*.^12^, have demonstrated the benefits of the buried Mie resonator arrays on antireflection, focusing on the contribution of the strongly substrate-coupled Mie resonances. Here, we present entirely new discoveries of their antireflection behaviors and give a comprehensive understanding of the antireflection structure. There are mainly two competing mechanisms contributing to the antireflection effects in the buried Mie resonator arrays with a specific period: one is the strong forward scattering from Mie resonances, which dominates at the long wavelength; the other is the scattering modulated interference antireflection that dominates at the short wavelength and with the position of the reflectance minimum deviating from the ideal destructive interference condition. We manipulate both antireflection effects by simply mediating the thickness of the dielectric cover layer on the short silicon nanostructures to simultaneously realize an excellent broadband antireflection and a low carrier recombination for photovoltaic applications. We have further combined the dielectric layers with an optimized multi-scale textures (nanostructures are optimally formed on micron-scale pyramids) to minimize the reflectance (the *R*_ave_ reaches as low as 2.43% over the wavelength from 400 to 1100 nm) and the recombination, and hence have successfully realized an independently certified conversion efficiency (*η*) of 18.47% for the nanostructured silicon solar cells on a large-size wafer (156 mm × 156 mm).

## Results

### Enhanced Mie resonances and interference effects from SiN_x_ layers

**Figure 1:**
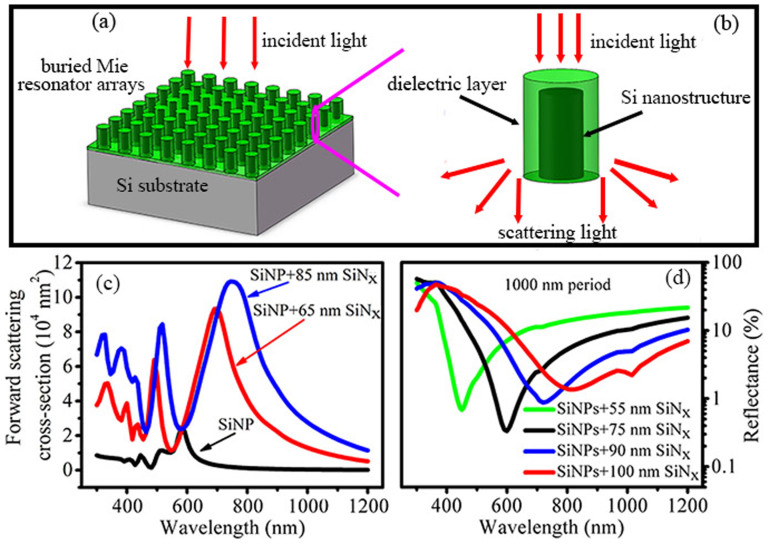
Schematic diagram of (a) the buried Mie resonator arrays and (b) the composition of a single buried Mie resonator together with its interaction with incident light. (c) Forward scattering cross-section for a single SiNP without SiN_x_ layer and with 65 nm and 85 nm SiN_x_ coating. The SiNP has a radius of 75 nm and height of 100 nm. (d) Calculated reflectance of the 1000 nm periodic SiNPs buried by SiN_x_ layers of varying thickness.

Figure 1c shows the forward scattering cross-sections for a single silicon nanopillar (SiNP) without and with SiN_x_ coatings calculated by finite-difference time-domain (FDTD) method. The forward scattering spectrum of the SiNP exhibits two scattering peaks, which are similar to the previous study and are attributed to the Mie resonances^12^. The lower-order Mie resonance mode shifts from 582 to 691 nm and further to 754 nm, when the SiNP are coated with 65 and 85 nm SiN_x_, respectively. Another prominent feature for the SiN_x_-layer-coated SiNP is the dramatically enhanced Mie scattering cross-section. And with the increase of the SiN_x_ layer thickness, the forward scattering is gradually enhanced with the peak shifting to the longer wavelength. It is obvious that the coated SiNP plays a role as Mie resonator and we can manipulate its resonance properties (including resonance wavelength and intensity) by simply regulating the thickness of the SiN_x_ covering layer, which is different from previous studies where the SiNP diameter was adjusted^13^,^22^,^23^. Such core-shell structures are also widely adopted in plasmon resonances due to the fact that they provide more parameters to manipulate the light scattering^24^. It is anticipated that the strong forward scattering light of the buried Mie resonators, shown in Figure 1b, will greatly contribute to suppressing the surface reflection when introduced to a silicon substrate.

Before confirming that, we present another antireflection effect in the buried Mie resonator arrays. A buried Mie resonator array reduces to a dielectric-covered planar surface when its period is infinitely large, so we can anticipate that an array with a large period may partly exhibit the well known interference antireflection effect of a coated planar surface. To demonstrate this effect, we have carefully studied the reflectance spectra of the buried SiNP arrays (SiNPs) with a period of 1000 nm by varying the thickness of the SiN_x_ layer, which is plotted in Figure 1d. Remarkable reflectance dips occur around the wavelengths satisfying the destructive interference formula: n*d = *λ*/4, where *n* is the refractive index of the SiN_x_, *d* is the layer thickness and *λ* represents the incident wavelength. Therefore, these reflectance dips should be attributed to the interference antireflection effect from the SiN_x_ layers. In the figure, we can also observe that the minimal reflectance of the 75-nm-SiN_x_-layer-buried SiNPs is the lowest as compared to those of the other three. On the one hand, it is relative with the optimal matching of the refractive index which enables to realize completely destructive interference (note that the refractive index of silicon depends on the wavelength, and thereby it is possible to make *n* = 2 become the optimal refractive index value at a certain wavelength, more detailed explanation see Figure S1); On the other hand, as the thickness of the SiN_x_ layer increasing to >75 nm, the light scattering is enhanced and thus destroys the interference antireflection effect to some extent, resulting in the increased minimal reflectance value. In fact, due to the enhanced scattering, the minimal reflectance of a silicon wafer with 75-nm-SiN_x_-layer-buried SiNPs on the top is indeed higher than that of a planar wafer only with a 75 nm SiN_x_ coating (see Figure S1).

**Figure 2:**
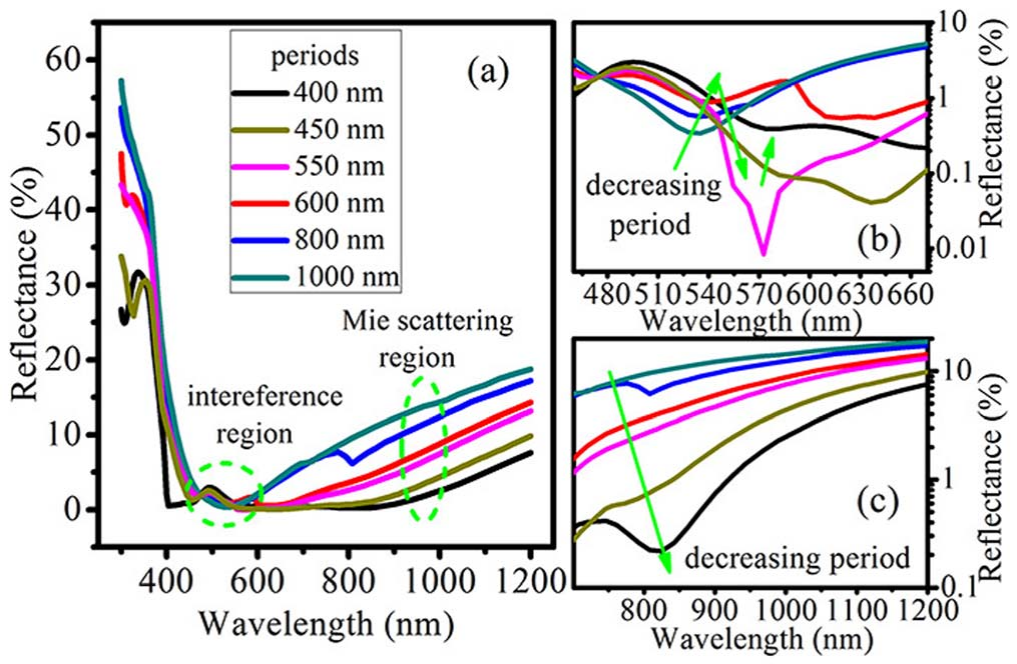
(a) Calculated reflectance spectra of the SiN_x_-layer-buried SiNP arrays, where the thickness of the SiN_x_ layer is fixed at 65 nm and the period of SiNP arrays varies from 1000 to 400 nm. The zoomed-in reflectance spectra of (b) the interference and (c) the Mie scattering regions indicated by two dashed circles in the Figure (a).

Figure 2a further shows that the reflectance curves approximately preserve the same shape when decreasing the periods of the buried SiNPs from 1000 nm to 400 nm, indicating that the interference antireflection effect is retained even in a rather dense SiNPs. The reduction of reflectance in the long wavelength region with decreasing SiNPs period originates from another antireflection mechanism, which will be discussed below. Figure 2b exhibits the amplified reflectance curves in the destructive interference region (indicated by dashed circle in Figure 2a) to obtain an insight into the influence of array density on interference antireflection. We can observe that the wavelength of the minimal reflectance gradually shifts to longer wavelength when decreasing the period from 1000 to 600 nm due to the increasing light scattering and interparticle interactions. When the period of the SiN_x_-layer-buried SiNP array decreases to 550 nm, the reflectance dip is changed dramatically with a relatively large red-shift of the reflectance minimum, which may be attributed to the strong interplay between the interference and the Rayleigh anomaly. With the period further decreasing to 400 nm, the wavelength of the minimal reflectance reaches 578 nm. Though it deviates from the wavelength determined by destructive interference formula, it should still be originated from the interference antireflection mechanism but modulated by light scattering and interparticle interactions.

### Antireflection mechanisms in the buried Mie resonator arrays

**Figure 3:**
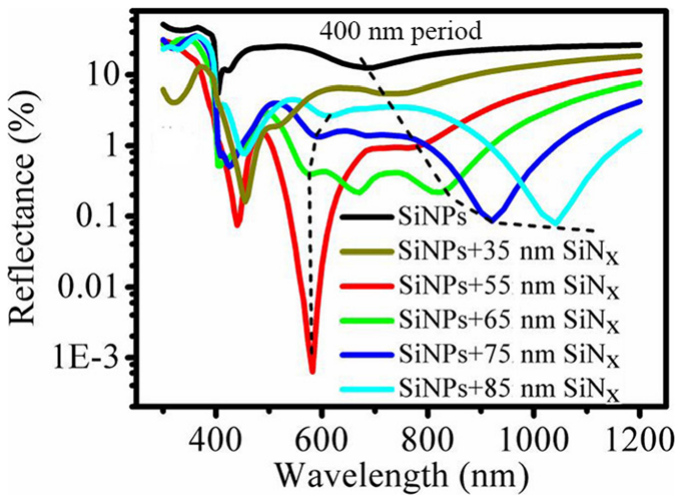
Calculated reflectance of the unburied and SiN_x_ film buried SiNP Mie resonator arrays. The period of the SiNPs is 400 nm. The thickness of the SiN_x_ layer varies from 35 nm to 85 nm. The dashed lines guide the reflectance dips for two different antireflection mechanisms.

Now, we turn to investigate the reflectance spectra of the 400-nm-periodic SiNPs standing on a silicon substrate and buried by the SiN_x_ layers with different thicknesses to comprehensively reveal the novel antireflection behaviors of the typical buried Mie resonator antireflection arrays. As guided by the dashed line in the region from 680 to 1100 nm in Figure 3, broad reflectance dips are evidently observed with their minimal values gradually declining, when the thickness of the SiN_x_ layer increases from 0 to 85 nm. Furthermore, the red-shift of the dips with increasing the SiN_x_ layer thickness is also in agreement with that observed for the Mie resonance in a single SiN_x_-layer-coated SiNP (see Figure 1c). Therefore, we attribute the reflectance dip in the long wavelength range to the forward Mie scattering, and its decline is caused by the enhanced Mie resonance. In the previous studies, the Mie resonant scattering is actually discerned as the reason for the reflectance dip of the unburied SiNPs^22^. However, it should be noted that the resonant properties are hard to be quantitatively analyzed by the classical Mie theory due to the influence of the substrate and interparticle interaction^12^,^22^,^24^,^25^. Figure 2c presents that the Mie resonance is enhanced with the resonant wavelength red-shifting when decreasing the buried SiNPs period. Hence, besides the SiN_x_ coating, the Mie resonant properties in buried SiNPs are also affected by array density.

From Figure 3, we can observe another reflectance dips outside the Mie resonance region at the wavelength around 600 nm, also guided by a dashed line (It is worth mentioning that the sharp decreases of reflectance around 400 nm are explained by the Rayleigh anomaly from the periodic arrays). When increasing the SiN_x_ layer thickness from 55 to 85 nm, the reflectance dips rise and shift from 573 to 609 nm, similar to that observed in Figure 1d. Clearly, the interference antireflection effect from the SiN_x_ layer dominates other antireflection mechanisms at this wavelength range, and results in these reflectance dips. However, unlike that in the 1000-nm-periodic SiN_x_-layer-buried SiNPs, the minimal reflectance does not happen at the wavelength where the destructive interference condition is met, because of the remarkably increased influence from light scattering and interparticle interaction in the relatively dense buried SiNPs, as discussed before. The increased minimal reflectance value with increasing SiN_x_ layer thickness also originates from both the enhanced light scattering, which is the more important factor in the case, and the deviation of the optimal refractive index matching.

From the discussion above, it is evident that there are two antireflection mechanisms for the SiN_x_-layer-buried SiNP Mie resonator arrays with a given period: one is the enhanced Mie resonant scattering, which mainly dominates the antireflection mechanisms at the long wavelength (>680 nm); the other is the scattering modulated interference antireflection, which dominates the mechanisms at the short wavelength (around 600 nm). And these two mechanisms are competing: with increasing SiN_x_ layer thickness (>55 nm), the interference antireflection weakens but the antireflection effect due to the forward Mie scattering is enhanced. Therefore, an excellent antireflection effect over a broad wavelength, and thus the lowest *R*_ave_, can be achieved in a relatively dense SiNP array by rationally designing the thickness of the SiN_x_ layer. Here, the optimal thickness of the SiN_x_ layer is 65 nm, and the *R*_ave_ for the corresponding SiN_x_-layer-buried SiNPs is 1.07% over the wavelength from 400 to 1100 nm, much lower than that of the SiNPs without SiN_x_ coating (19.49%).

**Figure 4:**
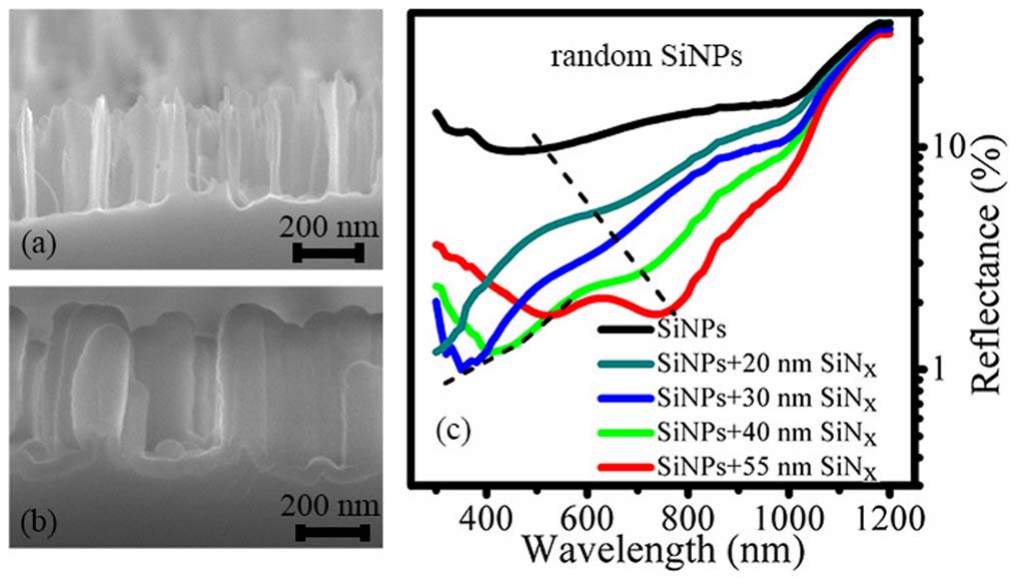
(a) SEM image of the SiNPs fabricated by MACE method. (b) SEM image of the SiN_x_-layer-buried SiNPs. (c) Experimental reflectance for both the unburied and SiN_x_-layer-buried random SiNPs atop planar silicon surfaces. The thickness of the SiN_x_ layer varies from 20 nm to 55 nm.

The antireflection mechanisms of the SiN_x_ layer buried SiNP Mie resonator arrays have also been examined in experiments, exhibited in Figure 4. The SiNPs are formed on the planar silicon surface by the metal assisted chemical etching (MACE) method. An scanning electron microscopy (SEM) image shows that they are non-periodic with a height of about 350 nm (Figure 4a). Their surfaces are conformally coated with a SiN_x_ layer via plasma enhanced chemical vapor deposition (PECVD), as shown in the Figure 4b, from which the thickness of the SiN_x_ layer is also estimated. Figure 4c depicts the measured reflectance of both the SiN_x_-layer-buried (with different thickness) and the unburied SiNPs varying with the wavelength. Similar to the results by the FDTD calculation, the reflectance of the SiN_x_-layer-buried SiNPs is much lower than that of the pure SiNPs over a broad wavelength. Also, there are two reflectance dips for the SiN_x_-layer-buried SiNPs (guided by dashed lines), and the reflectance value of the dip at the short wavelength increases, while that at the long wavelength decreases as the thickness of the SiN_x_ layer is increased from 30 to 55 nm. Likewise, we attribute these two reflectance dips to the modulated interference antireflection and the Mie resonance, respectively. When the thickness of the SiN_x_ layer is 55 nm, both antireflection mechanisms function effectively, leading to a low reflectance over a broad wavelength and thus the lowest *R*_ave_ (the *R*_ave_ are 13.54%, 8.57%, 6.77%, 5.21% and 4.09% for the SiNPs without SiN_x_ and with 20, 30, 40, 55 nm SiN_x_ coating over the wavelength from 400 to 1100 nm, respectively). It should be noted that though the SiNPs are random in the experiment and their dimensions are not identical to that in the simulation, their antireflection behaviors with respect to the thickness of the SiN_x_ layer are quite similar to the periodic arrays calculated by FDTD, demonstrating the validity of the explanation and universality of the antireflection effects.

### Benefits from the buried Mie resonator arrays

**Figure 5:**
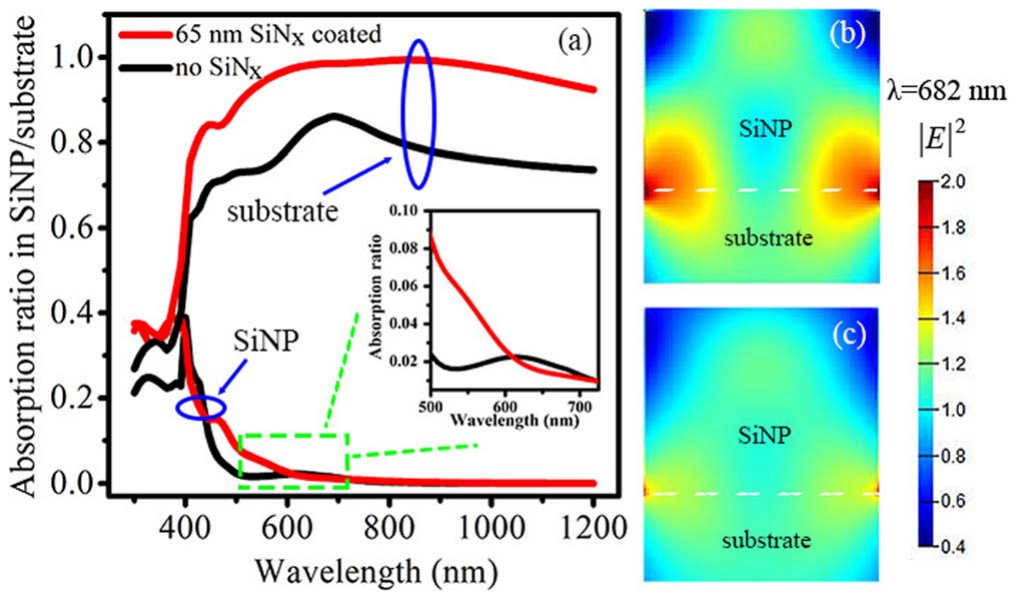
(a) Calculated absorption ratio within a single SiNP and the substrate as a function of wavelength. The inset shows a schematic diagram of a nanostructured silicon wafer as well as the magnified absorption spectra within the single SiNP over the wavelength from 500 to 730 nm. (b) Electric field distribution in a cross-section within a single unburied SiNP. (c) Electric field distribution in a cross-section within a single 65-nm-SiN_x_-film-buried SiNP. The dashed white lines outline the interfaces between the substrate and the SiNP.

In order to provide a further insight into the function of the buried Mie resonator arrays, the light absorptions within a single SiNP are calculated by FDTD for both the SiN_x_-layer-buried and the unburied SiNPs, illustrated in Figure 5a. Also shown are the absorption spectra in the substrate, which are calculated following the equation: *α*_S_ = 1 − *R* − *α*_NP_, where *R* is the surface reflectance, *α*_S_ and *α*_NP_ denote the absorption ratio in the silicon substrate and SiNP, respectively. There is an absorption peak in the single unburied SiNP in the wavelength from 550 to 730 nm (see the inset in Figure 5a) due to the strongly confined cavity mode in the single SiNP (shown in Figure 5b). Nevertheless, no absorption peak is observed in a single 65-nm-SiN_x_-layer-buried SiNP because the cavity mode is significantly attenuated, as exhibited in Figure 5c. In fact, even in the wavelength where the strong Mie scattering happens, the cavity mode in a single SiN_x_-layer-buried SiNP is also quite faint (see Figure S2). As a result, the light absorption within a single SiN_x_-layer-buried SiNP is almost identical to that in the single unburied SiNP except a slight increase in the short wavelength, despite its remarkably decreased surface reflectance. Regarding the absorption within the substrate, it is distinctly higher for that with the 65-nm-SiN_x_-layer-buried SiNPs as compared to that with pure SiNPs over a broad wavelength from 450 to 1200 nm.

**Figure 6:**
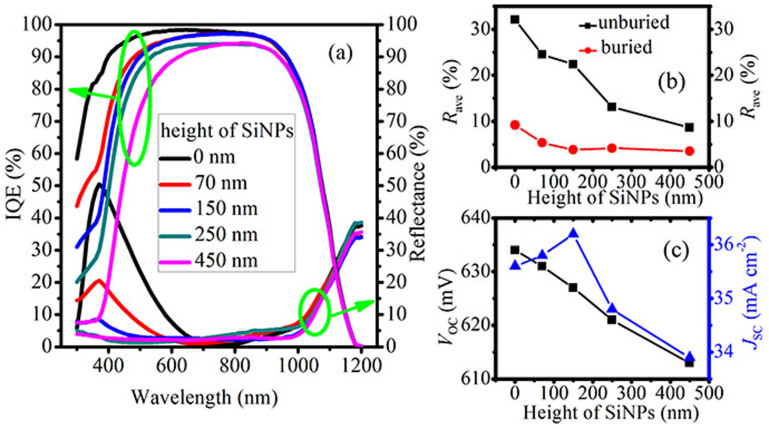
(a) Experimental reflectance and IQEs of the buried SiNPs versus the wavelength, (b) *R*_ave_ of the buried and unburied SiNPs over the wavelength from 400 to 1100 nm, (c) *V*_OC_ and *J*_SC_ of the buried SiNPs. The nanostructure height varies from 0 to 450 nm.

In the previous studies, the reflectance of silicon nanostructure arrays has a substantial dependence on nanostructure height^26^,^27^. Here, we experimentally compare the reflectance spectra of the SiN_x_-layer-buried SiNPs with nanostructure height from 0 to 450 nm to reveal its influence on the antireflection properties. As shown in Figure 6a, the antireflection effect of the planar sample (i.e. the 0 nm case) depends strongly on the wavelength. In the destructive interference region (600–900 nm), the surface reflectance is suppressed, while the reflectance increases drastically outside of this region. On the contrary, when the nanostructure height is higher than 150 nm, an excellent broadband antireflection is achieved due to the combined antireflection from the destructive interference and the Mie resonance as discussed above, leading to the much lower *R*_ave_, shown in Figure 6b. In addition, the *R*_ave_ of the 150 nm SiN_x_-layer-buried SiNPs is comparable to that of the 450 nm SiN_x_-layer-buried SiNPs, exhibiting a weak dependence on nanostructure height, which is totally different from that of the unburied SiNPs. Therefore, we can conclude that the reflectance of the buried Mie resonator arrays is less sensitive to the nanostructure height when higher than 150 nm.

The result is exciting for photovoltaic applications, since it enables us to reduce the nanostructure height without sacrificing the optical properties. As it is known, the entire silicon nanostructure layer is heavily doped in a diffused nanostructured wafer. Therefore, with respect to the electrical properties, the reduction of nanostructure height will not only result in lower surface recombination due to the lower surface area, but also prominently contribute to lowering the Auger recombination due to the reduction of the heavily doped emitter volume^27^. These results will further lead to higher internal quantum efficiency (IQE) in the short wavelength, as shown in Figure 5a, because the short-wavelength photons are mainly absorbed near the wafer surface. Figure 6c presents the open circuit voltage (*V*_OC_) and the short circuit current density (*J*_SC_) of the solar cells with SiN_x_-layer-buried SiNPs on the surface. As the nanostructure height increases, the *V*_OC_ declines due to the increased carrier recombination, and the *J*_SC_ increases first due to the dominance of the reduced surface reflectance and then decreases owing to the deterioration of IQE while the reflectance almost stays unchanged. As a consequence, the solar cell with 150 nm SiN_x_-layer-buried SiNPs atop the planar silicon surface has the highest *η* (the *η* are 17.9%, 18.0%, 18.1%, 17.2% and 16.2% for the solar cells with nanostructure height of 0, 70, 150, 250 and 450 nm, respectively). Our results demonstrate that a relatively low carrier recombination and reflectance can be simultaneously achieved in the silicon solar cells with dielectric-layer-buried Mie resonator arrays benefiting from the weak dependence of the antireflection on nanostructure height (in a certain range), thus resulting in a high *η*.

### Combine dielectric layer with optimized multi-scale textures for high-efficiency solar cells

**Figure 7:**
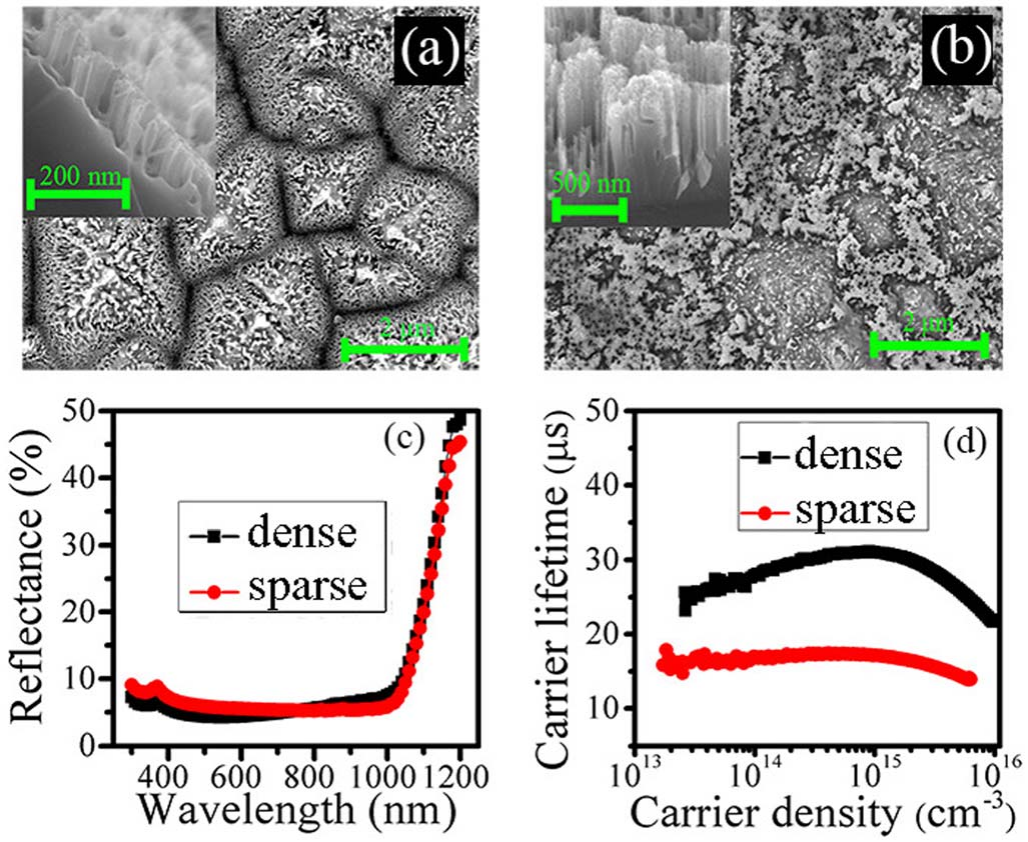
Plan-view SEM images of the (a) dense and (b) sparse multi-scale textures. The insets are the cross-sectional SEM images of the corresponding silicon nanostructures. Comparisons of (c) experimental reflectance and (d) carrier lifetime between these two multi-scale textures.

Beside planar surface, we have also studied the dielectric-layer-buried Mie resonator arrays on micron-scale structures—for instance the SiNP Mie resonator arrays in Figure 7 are formed on micron-scale pyramid surfaces forming multi-scale textures and then buried by dielectric layers (buried multi-scale textures for short). It was reported that multi-scale textures have the ability to significantly diminish the surface recombination by reducing the nanostructure height, while an ultra-low surface reflection is maintained due to the combined antireflection effects from the silicon nanostructures and the micron-scale pyramidal texture^28^,^29^. Hence the multi-scale textures are believed to be an effective technique for realizing high-efficiency solar cells^30^. Here, we focus on the influence of the surface morphology of the multi-scale textures on the optical and electrical properties of the solar cells (in this work, the surface morphology is controlled by the concentration of H_2_O_2_ contained in the MACE solution). Figure 7a,b contrastively shows two different morphologies of multi-scale textures: one with rather dense SiNPs uniformly distributed on the whole pyramids (the dense multi-scale textures); the other one with the SiNPs only on the bottom regions of the pyramids but bare at the top regions (the sparse multi-scale textures). Figure 7c presents that the surface reflectance of the dense multi-scale textures is comparable to that of the sparse one over the whole wavelength, even if its nanostructure height is much lower (the nanostructure heights are 150 and 600 nm for the dense and sparse ones, respectively, see the inset of Figure 6). Regarding the electrical properties, it is exciting to find that the effective minority carrier lifetime of the dense multi-scale textures is much higher than that of the sparse one, as illustrated in Figure 7d, which can be attributed to its much lower surface area^31^. These results demonstrate that both excellent antireflection and low carrier recombination can be attained in the dense multi-scale textures.

**Figure 8:**
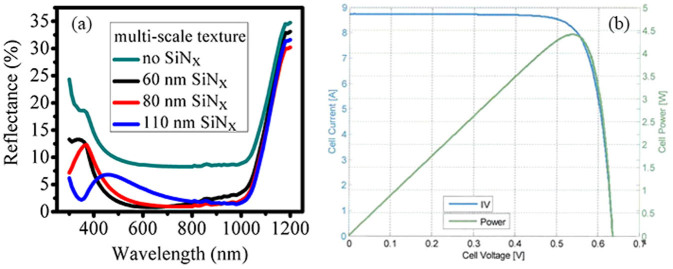
(a) Experimental reflectance of both the unburied and SiN_x_-film-buried multi-scale textures, in which the thickness of the SiN_x_ film varies from 60 to 110 nm. (b) Current-voltage characteristic of the 18.47%-efficient multi-scale textured silicon solar cell, which is independently measured by the TÜV Rheinland Co., Ltd., official Test Report No. 15067482.001.

Based on the optimized surface morphology, we have further thoroughly investigated the reflectance properties of the SiN_x_-layer-buried multi-scale textures in experiments (here, the nanostructure height is controlled to be 100 nm). Just like the reflectance behaviors on a planar surface, Figure 8a shows that the reflectance of the SiN_x_-layer-buried multi-scale textures is remarkably reduced relative to that of the multi-scale textures without SiN_x_ coating, which is ascribed to the enhanced antireflection effects from the SiN_x_ layer. When the multi-scale textures are buried by an 80 nm SiN_x_ film, an ultra-low reflectance over a broad wavelength (500–1000 nm) is achieved due to the best compromise of both the strong interference antireflection and forward scattering, resulting in the lowest *R*_ave_ of 2.43% over the wavelength from 400 to 1100 nm. It is worth mentioning here that the ineffectiveness of the reflectance reduction by the 110-nm-SiN_x_-layer-buried multi-scale textures for wavelength larger than 1000 nm is probably due to the weak absorption of silicon in the wavelength range together with the thin wafer thickness of only 180 μm (in this case, the reflectance has no much relationship with the antireflection effects on the front surface, such as the forward scattering, due to the fact that the incident light can be easily reflected back from the back surface). Based on the optimized SiN_x_-layer-buried multi-scale textures, we have successfully achieved an *η* of 18.47% (equal to the maximum power of 4.414 W) for the large-scale nanostructured silicon solar cells with an area of 238.95 cm^2^ (156 mm × 156 mm), which is independently certified by the TÜV Rheinland Co., Ltd. and exhibited in Figure 8b. Comparing to the results in the Figure 5b, where the SiNPs are fabricated on the planar surfaces, a dramatic improvement in efficiency for the solar cells with the buried multi-scale textures benefits from the much lower carrier recombination (owing to the shorter silicon nanostructures), together with better antireflection effect.

## Discussion

Figure 3 and 4c explicitly present two competing antireflection behaviors in the buried Mie resonator arrays, which are composed of silicon nanostructures atop a silicon substrate and buried by dielectric films. As the dielectric layer thickness increasing, the Mie resonant scattering that dominates the antireflection mechanisms at the long wavelength is enhanced, while the interference antireflection effect is attenuated, which is the dominant mechanism at the short wavelength. The discovery of these two competing antireflection mechanisms enables us to achieve a superior broadband antireflection effect by the rational design of the buried Mie resonator arrays. Meanwhile, it should be noted that both the interference antireflection and the Mie resonance are influenced by array density. Figure 5 further demonstrates that the reduced reflection mainly contributes to the absorption in the silicon substrate rather than in the silicon nanostructures, indicating that the buried Mie resonator arrays primarily act as a transparent antireflection layer. It should be pointed out that this is beneficial for silicon solar cells since the SiNPs layer often serves as a “dead layer” in terms of electrical properties^32^. The design of the buried Mie resonator arrays enables us to loosen the requirement on the nanostructure height to reduce significantly the carrier recombination while retaining the optical properties. We have also incorporated Mie resonator arrays into the multi-scale textures, but focus on demonstrating the influence of their surface morphology on both the optical and electrical properties. Figure 7 shows that the comparatively dense Mie resonator arrays are beneficial to obtain a low reflectance and a low carrier recombination. Through optimizing the buried multi-scale textures, the reflection is largely suppressed, especially over the wavelength from 500 to1000 nm, and thus we have successfully obtained an independently certified *η* of 18.47% for nanostructured silicon solar cells on a large-size wafer (156 mm × 156 mm). It is worth mentioning that though similar antireflection structures have been reported^12^,^33^, including in our previous paper^32^, the overall understanding of the buried Mie resonator arrays is still quite deficient (such as the antireflection mechanism and influence of surface morphology), thus not achieving the best optical and electrical properties. Here, our presented understanding of the buried Mie resonator arrays can be beneficial for the development of high-performance optoelectronics such as photovoltaics and photodetectors.

## Methods

### Numerical calculation

We employ the FDTD method to study the optical properties of both a single SiNP with circular cross-section and periodic SiNPs covered by SiN_x_ layers with varing thicknesses (including the case without coating). The SiNP has a radius of 75 nm and height of 100 nm. The light source is a plane wave with a wavelength ranging from 300–1200 nm and normal incidence to the substrate (Figure 1a) or axial incidence to the single SiNP (Figure 1b). For calculating the forward scattering cross-section spectra of a single SiNP, the simulations are performed in boxes of 5 μm × 5 μm × 1.2 μm with perfectly matched layer boundaries and mesh grid of 2 nm. The scattering powers are obtained by means of frequency-domain transmission monitors located in the scattered field region. In the determination of the reflectance spectra of the periodic SiNPs atop a silicon substrate, the simulation boxes are *p* × *p* × 800 nm with periodic boundary conditions in the in-plane directions, where *p* is the array pitch. The bottom sides of the substrates are outside of the simulation region so that the substrate can be regarded as infinite thick. The mesh grid is also set to be 2 nm. All the above simulations are performed using a commercial software package (FDTD Solutions v8, Lumerical 2013), from which the optical constants of Si are directly extracted. The refractive indexes of SiN_x_ are set to be a constant value of 2 without considering the extinction coefficient.

### Experimental details

In the experiments, the used silicon wafers are pseudo-square (156 mm × 156 mm) p-type Czochralski crystalline silicon with a thickness of 180 μm and resistivity of 1–3 Ω·cm. SiNPs are formed on one side of either the polished or the micron pyramidal textured Si wafers by MACE method^26^,^32^. After that, these wafers are dipped into 40 vol% nitric acid aqueous solution to remove the metal residuals, and then are immersed in the diluted HF solution to remove silicon oxide layers. Subsequently, these cleaned nanostructured wafers undergo a standard solar cell manufacturing process, namely n-type diffusions on the nanostructured sides using POCl_3_ as the dopant source (the sheet resistances are 80 Ω/□), edge junction isolation by ion etching, removal of the phosphosilicate glass through HF etching, deposition of SiN_x_ layers by PECVD as well as the fabrication of front and back electrodes by screen printing technique. Note that for the experimental investigation of the reflectance of the SiN_x_-layer-buried SiNPs, the thicknesses of the SiN_x_ layers are controlled by the deposition time.

### Characterization

The microstructures of the SiNPs both with and without the SiN_x_ coatings are investigated by field emission scanning electron microscopy (FE-SEM, FEI Sirion 200). The effective carrier lifetime of the nanostructured silicon wafers (treated with nanostructure texture on double sides and symmetrical passivation by SiN_x_:H layers) are measured using quasi-steady state photoconductance decay method in WCT-120 instrument (Sinton). The IQEs together with the surface reflectance as a function of wavelength are determined in the equipment of QEX10 (PV Measurements). The current-voltage tester is used to characterize the electrical performances of the solar cells under AM1.5 spectrum at the temperature of 25°C. Therein, the current-voltage characteristic of the highest nanostructured solar cell is independently confirmed by the TÜV Rheinland Co., Ltd.

## Author Contributions

S.Z. carried out the simulations and experiments. S.Z., Y.Z., Z.H. and W.S. contributed to the data analysis and figures. S.Z. wrote the main manuscript text. W.S. reviewed the manuscript.

## Supplementary Material

Supplementary Information supporting information
