# Supplementary material for: Superior broadband antireflection from buried Mie resonator arrays for high-efficiency photovoltaics
Source: Sci Rep. 2015 Mar 9;5:8915. doi: 10.1038/srep08915 (PMC4352919; doi:10.1038/srep08915)
Supplement: Supplementary Information — supporting information [file srep08915-s1.pdf]

Supporting information

Superior broadband antireflection from buried Mie resonator arrays for high-efficiency photovoltaics

Sihua Zhong, Yang Zeng, Zengguang Huang and Wenzhong Shen

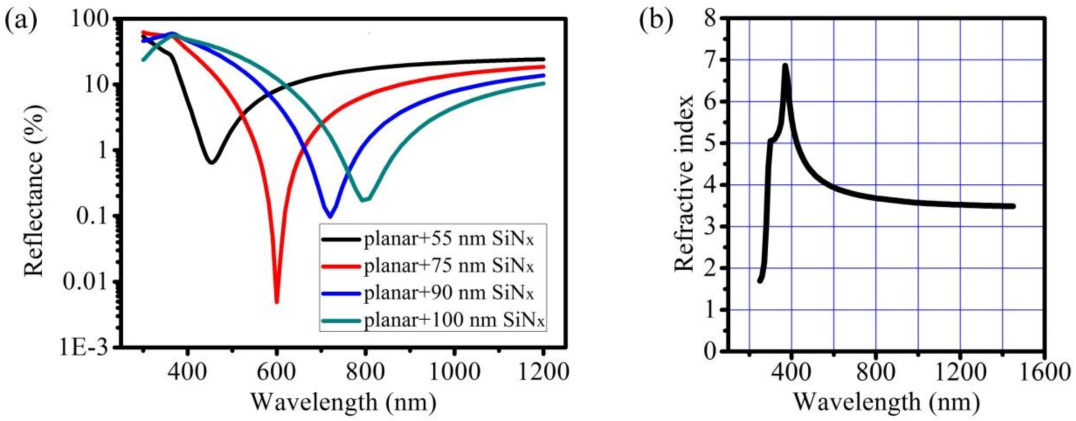

Figure S1 (a) Surface reflectance of the planar silicon covered by SiN_x layers with different thicknesses. (b) Refractive index of silicon as a function of wavelength.

For the SiN_x layer coated planar substrate, the minimal reflectance happens strictly at the wavelength where the destructive interference condition is satisfied:

$$n * d = \lambda / 4 \tag{1}$$

where n is the refractive index of the SiN_x (the value is set to 2 in the simulation), d is the layer thickness and λ represents the incident wavelength. To reach completely destructive interference, n should satisfy:

$$n=\sqrt{n_s * n_0} \tag{2}$$

where n_s is the refractive index of the silicon substrate, n_0 is the refractive index of air (the value is 1). As we know, the refractive index of silicon depends on the incident wavelength and approaches 4 near the wavelength of 600 nm, shown in the Figure S1b. Therefore, the completely destructive interference condition is satisfied when the planar silicon is covered with 75 nm SiN_x film, thus reaching the lowest reflectance value as compared to the other three.

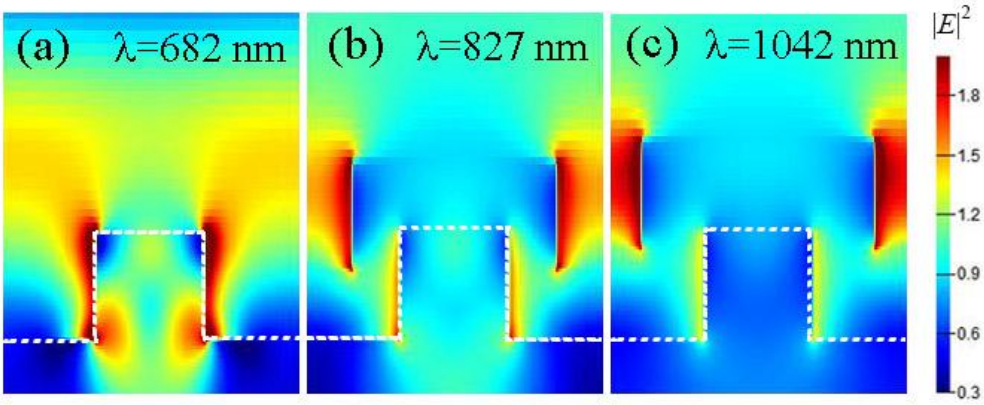

Figure S2 Electric field intensities in a cross-section for a single (a) unburied, (b) 65 nm and (c) 85 nm SiNx layer buried SiNP under the incident lights where Mie resonances happen. The dashed white line outlines the interface between silicon and air (or SiN_x).

From the figure, we can observe that the optical mode within the SiNP is greatly attenuated with the thickness of the SiN_x layer increasing. On the contrary, the electric field in the substrate is greatly enhanced except in the region below the SiNP, demonstrating the increased light scattering.
